# Sophora tonkinensis enhances activation of cGAS-STING pathway and restrains HBV replication

**DOI:** 10.3389/fphar.2025.1630460

**Published:** 2025-10-13

**Authors:** Yuanyuan Guo, Jincai Wen, Xueting Wang, Xianling Wang, Yingjie Xu, Siqi Huang, Zongliang Lu, Xiaoyan Chen, Ang Huang, Zhijie Ma, Junling Cao, Xiaoyan Zhan, Zhaofang Bai

**Affiliations:** ^1^ School of Chinese Materia Medica, Beijing University of Chinese Medicine, Beijing, China; ^2^ Senior Department of Hepatology, Chinese PLA General Hospital, Beijing, China; ^3^ School of Pharmacy, Chengdu University of Traditional Chinese Medicine, Chengdu, China; ^4^ School of Medicine, Nankai University, Tianjin, China; ^5^ Senior Department of Gastroenterology and Hepatology, Chinese PLA General Hospital, Beijing, China; ^6^ Department of Pharmacy, Beijing Ditan Hospital, Capital Medical University, Beijing, China; ^7^ Department of Pharmacy, Dongfang Hospital, Beijing University of Traditional Chinese Medicine, Beijing, China; ^8^ Caojunling National Veteran Pharmaceutical Worker Inheritance Studio, Beijing, China

**Keywords:** Sophora tonkinensis gagnep. [fabaceae], HBV, cGAS-STING pathway, antiviral response, immonology

## Abstract

**Background:**

Cyclic GMP–AMP synthase (cGAS)-Stimulator of interferon genes (STING) signaling pathway plays a vital role in innate immune response. Once activated, cGAS-STING pathway mediates the production of type I IFNs and pro-inflammatory cytokines, triggering antiviral response. The Chinese medicine *Sophora Tonkinensis* Gagnep. is a commonly used traditional Chinese medicine with the effects of clearing away heat and detoxification, subduing swelling and relieving pharynx. Modern studies have shown that it has antiviral activity, however, its mechanism of action is still not clear.

**Methods:**

In this study, we used the botanical drug Sophora tonkinensis Gagnep. (Fabaceae) and investigated the effect of *Sophorae tonkinensis* extract (STE) on the activation of the cGAS-STING pathway in BMDMs and THP-1 cells, the mechanism by which STE regulates cGAS-STING pathway were studied. We also evaluated the antiviral activity of STE in an HBV mouse model by hydrodynamic injection of pAAV-HBV1.2 plasmid. The content levels of HBsAg and HBeAg in the serum of mice were detected by Elisa, and the level of HBV-DNA was detected by PCR-Fluorescence Probing in One-Tube.

**Results:**

STE effectively promoted the activation of the cGAS-STING pathway in BMDMs and THP-1, but had no effect on cytoplasmic RNA-induced RIG-I signaling activation. Furthermore, STE can be effective, promoting 2′3′-CGAMP synthesis. Importantly, STE could effectively restrain HBV replication and promote cGAS-STING pathway activation in HBV mouse model.

**Conclusion:**

STE could effectively promote the activation of cGAS-STING pathway by facilitating cGAMP synthesis by cGAS, exhibiting obviously inhibitory effect on HBV replication. STE might serve as an effective therapeutic approach against viral infections diseases.

## 1 Introduction

cGAS-STING is an important innate pathway recognizing cytosolic DNA and triggering immune response. Upon binding to dsDNA, cGAS is activated and then catalyzes ATP and GTP into 2′3′-cGAMP, which in turn activates STING, STING then undergoes oligomerization and transports to the Golgi (Su et al., 2020; Zheng et al., 2024; Luo et al., 2023; Li et al., 2021), where it assembles the signalosome with tank-binding kinase 1 (TBK1) and the transcription factor interferon (IFN) regulatory factor 3 (IRF3), resulting in IRF3 phosphorylation by TBK1 and then the transcription of type I IFNs (Wen et al., 2023; Luo et al., 2024; Hui et al., 2024). Type I IFNs bind to its receptor and activates JAK-STAT pathway, which in turn induces the transcription of IFN-stimulated genes (ISGs) The ISG transcription proteins, such as ligoadenylate synthetase (OAS), cholesterol 25-hydroxylase (CH25H) and myxovirus resistance (Mx), exert various antiviral functions. cGAS-STING pathway has been reported to play crucial roles in virus infection (Qin et al., 2024; Dunphy et al., 2018).

cGAS-STING pathway has been reported to be involved in the recognition of numerous DNA virus. For example, it has been revealed that activation of the cGAS-STING pathway impedes herpes simplex virus 1 (HSV-1) replication in mice microglial cells (Reinert et al., 2016). Moreover, cGAS-STING pathway induces type I IFN production and then suppresses replication of cytomegalovirus (CMV) (Lio et al., 2016). HBV nucleic acids in intracellular can be sensed by cGAS proteins and then activate the downstream signaling, STING agonists effectively increases type I IFN and inhibits HBV repliaction (Zhao L. et al., 2023; Dansako et al., 2016; Huérfano et al., 2022). Previous studies have established that TRIM29 plays a critical regulatory role in the innate immune response mediated by the cGAS–STING pathway. As an E3 ubiquitin ligase, TRIM29 attenuates the duration and intensity of STING signaling by facilitating K48-linked ubiquitination and subsequent proteasomal degradation of STING, thereby preventing excessive or prolonged immune activation (Xing et al., 2017). Furthermore, TRIM29 has been implicated in suppressing the production of antiviral type I interferon through mechanisms that may involve the enhancement of PEAK1-mediated endoplasmic reticulum stress responses and ROS-induced oxidation of TBK1, although these pathways require further validation (Wang J. et al., 2024). Instead, TRIM18 (also known as MID1) functions as a negative regulator of TBK1. It recruits the protein phosphatase PPM1A to dephosphorylate and inactivate TBK1, thereby inhibiting the interaction between TBK1 and its upstream adaptors Mavs and Sting, and the gene knockout of trim18 enhances TBK1-mediated antiviral signaling, which may contribute to the development of anti-viral drugs, and enhanced type I interferon response to RNA and DNA viruses (Fang et al., 2022). Therefore, enhancing cGAS-STING pathway activation may be an effective strategy to restrain viral infection.

The Chinese herb Sophorae tonkinensis is a traditional heat-clearing and detoxifying medicine with a variety of pharmacological activities including anti-tumor, anti-inflammatory, immunomodulatory, anti-viral, and hepatoprotective (ShuoWang et al., 2021). Pharmacological studies indicate that ST alkaloids exert anti-tumour effects by activating caspase-3 and inducing apoptosis in several cancer cell lines (Li et al., 2009). ST polysaccharides have also been found to have good hepatoprotective effects and can effectively reduce liver injury induced by CCl4 or acetaminophen and decrease the expression of ALT good AST (Zhang et al., 2021). Some clinical ST preparations have also been shown to have better hepatoprotective effects in patients with chronic hepatitis B. However, the antiviral function and mechanism of action of ST remains to be elucidated (Chen et al., 2017; Zeng et al., 2025).

In this work, we found that Sophorae tonkinensis extract (STE) could promote the activation of cGAS-STING by facilitating the synthesis of 2′3′-cGAMP, accompanied by elevated levels of interferon and pro-inflammatory factors, and also play an important role in inhibiting HBV replication in a mouse model of HBV. These findings suggest that active compounds from Sophora tonkinensis could serve as a promising lead for the development of new therapeutic agents against hepatitis B.

## 2 Materials and methods

### 2.1 Regents and antibodies

Dulbecco’s modified eagle medium (DMEM, PYG0073, Boster), Roswell park memorial institute 1640 medium (RPMI-1640, PYG0006, Boster), GenOpti medium (CT007.500, M&C gene technology ltd.), Fetal Bovine Serum (C04001-500, VivaCell), PBS (03.15018C, EallBio), M-CSF Protein, Mouse (HY-P7058, MedChemExpress), Polyinosinic-polycytidylic acid (Poly(I:C), HY-107202, MedChemExpress), Trizol (111003, Alcatel), VAMNE Magnetic Universal Total RNA Kit (ROA3301-01) Protease inhibitor cocktail (C0001, TargetMol), StarFect transfection reagent (C101-10, GenStar), StarScript III All-in-one RT (A230-10, GenStar), 2×RealStar Fast SYBR qPCR Mix (Low ROX) (A304-10, GenStar), Sodium chloride (C090701, YangGuangBio), IRF3 (phospho Ser396) antibody (GTX86691, Human/Mouse, 1:1000, GenTex), Anti-IRF3 (phospho S386) antibody (ab76493, Human 1:1000, Abcam), Anti-IRF3 antibody (ab68481, Human/Mouse 1:1000, Abcam), Hepatitis B Virus Core Antigen (ab8639, 1:100, Abcam), Hsp90 (5D6) Mouse mAb (251211, Human/Mouse, 1:1000, Zenbio), TMEM173/STING Polyclonal antibody (19851-1-AP, Human/Mouse, 1:2500, Proteintech), Universal Antibody Diluent (02.13451-100, EallBio) FITC anti-mouse CD3 (100204, Mouse, 1:200, BioLegend), PE anti-mouse CD4 (100408, Mouse,1:600, BioLegend), PerCP/Cyanine5.5 anti-mouse CD8a (100734, Mouse, 1:150, BioLegend), APC anti-mouse IFN-γ (505810, Mouse, 1:100, BioLegend), Zombie NIR™ Fixable Viability Kit (423105, 1:5000, BioLegend), 4% Paraformaldehyde Fix Solution (G1101-500ML, Servicebio), 2′3′-cGAMP ELISA Kit (501700, Cayman Chemical), LumiKine™ Xpress mIFN-β 2.0 (luex-mifnbv2, Mouse, InvivoGen), LumiKine™ Xpress hIFN-β 2.0 (luex-hifnbv2, Human, InvivoGen), Nuclear and Cytoplasmic Protein Extraction Kit (P0028, Beyotime). Human hepatitis B virus e antigen (HBeAg) ELISA Kit (ZC01803, Wantai biopharm), Human hepatitisB surface antigen (HBsAg) ELISA Kit (ZCY0503, Wantai biopharm), Quantitative PCR Diagnostic Kit for HBV-DNA (PCR-Fluorescence Probing in One-Tube) (B001, NaGene Diagnosis), ISD was synthesized as previously described by heating equimolar amounts of sense DNA and anti-DNA oligo acids to 95 °C for 5 min and then cooling to room temperature. The Sophora tonkinensis are derived from the dried roots and rhizomes of the legume *Sophora tonkinensis* Gagnep*.,* pAAV/HBV1.2 plasmid was from XJ Wang (Academy of Military Medical Sciences).

### 2.2 The quality control of STE

The botanical drug Sophorae Tonkinensis Radix et Rhizoma used in this study consists of the dried roots and rhizomes of Sophora tonkinensis Gagnep. (Fabaceae; batch no. 23011701, Taizhou Baicao Chinese Medicine Drinks Slice Co., Ltd.). The botanical material was excavated in Guangxi, China, in September 2022 and is deposited in the Department of Liver Diseases, Chinese PLA General Hospital, where it was authenticated as authentic by Professor Xiao-He Xiao. The crude drug appears as irregular, nearly circular slices 2–4 mm thick, with an outer surface brown to dark brown; the cortex is light brown and the xylem pale yellow in cross-section. It emits a characteristic bean-like odor and tastes extremely bitter (Heinrich et al., 2022).

For qualitative analysis of the active constituents in STE, reference standards of oxymatrine, oxysophocarpine, matrine, trifolirhizin, and maackiain were dissolved in methanol to prepare individual stock solutions. Weigh 2 g of STE precisely into a volumetric flask, add 20 mL of water, and prepare the test sample solution by sonicating for 60 min. Freeze-dry the solution. The test sample solution was prepared by accurately weighing 50 mg of lyophilized powder into a 10-mL volumetric flask, adding 8 mL of water, and sonicating for 30 min. After cooling to room temperature, the mixture was diluted to volume with water, mixed thoroughly, and filtered; the filtrate was used for analysis. Chromatographic separation was performed on a PFchrom EP C18 column (4.6 × 250 mm, 5 μm; FX-063) maintained at 35  °C. A gradient elution program was employed with acetonitrile and 4 mmol/L dipotassium hydrogen phosphate as mobile phases: 8%–10% acetonitrile (0–4 min), 10%–15% (4–15 min), 15%–27% (15–18 min), 27%–37% (18–30 min), 37%–75% (30–32 min), held at 75% (32–40 min), returned to 8% (40–41 min), and held at 8% until the end of the run. The flow rate was 1.0 mL/min, the detection wavelength was set at 215 nm, and the injection volume was 10 μL.

### 2.3 Animals

C57BL/6J male mice, weighing 18–20 g, aged 8 weeks, were supplied by Beijing SPF Biotechnology Co., Ltd. and were allowed to acclimatize for 1 week prior to the experiment. The mice were kept in a pathogen-free environment while being given adequate light and darkness as well as water and food. Animal experiments were permitted by the Animal Ethics Committee of the Fifth Medical Center of the General Hospital of the Chinese People’s Liberation Army (Approval Date ID: IACUC-2024-0006).

*In vivo* experiments, male C57BL/6J mice were constructed as HBV models by hydrodynamic injection of 20 μg pAAV-HBV1.2 plasmid. Different concentrations of STE (0.2 and 0.4 g/kg) or ETV (1 g/kg) were given daily (n = 6 per group), and the control group was injected with saline in the tail vein and given the same concentration of vehicle daily. It was given continuously for 4 weeks, and the serum levels of HBeAg, HBsAg and HBV-DNA in mice were measured weekly. The content levels of HBsAg (ZCY0503, Wantai biopharm) and HBeAg (ZC01803, Wantai biopharm) in the serum of mice were detected by Elisa, and the level of HBV-DNA was detected by PCR-Fluorescence Probing in One-Tube. The mice were executed at week 4, with serum, liver and spleen tissues collected (Sang et al., 2017).

### 2.4 Cells

BMDMs were derived from bone marrow of healthy wild-type mice and cultivated in DMEM (including 10% FBS and 1% P/S) with MCSF, and the BMDMs were seeded at a plate density of 1.2*10^6^ cells/mL, and planted in 12-well plates at 1 mL in each well. THP-1 (purchased from Procell, CL-0233) was purchased from Servicebio (BNCC358410) and cultured in 1640 at a seed plate density of 1.2*10^6^ cells/mL, with 0.5 mL per well planted in 24-well plates. HEK-293 (purchased from Procell, CL-0001) was cultured in medium including DMEM at a seed plate density of 5.5*10^5^ cells/mL, with 0.5 mL per well planted in 24-well plates (Wang et al., 2019).

BMDMs or THP-1 were first pretreated with various concentrations of STE or vehicle for 1 h, and then stimulated with ISD (2.5 μg/mL) or Poly(I:C) (2 μg/mL) for 2.5 h. Whole-cell lysates were collected for immunoblotting; the cells were lysed with Trizol at 4 h of stimulation with ISD or Poly(I:C), and the relevant genes were detected for transcription levels; at 8 h of stimulation with ISD or Poly(I:C), cell supernatants were collected to detect IFN-β levels. For plasmid transfection experiments, HEK-293 cells (24-well plate) were transfected with HA-cGAS (0.5 μg), HA-STING (0.5 μg), HA-TBK1 (0.5 μg), or HA-IRF3(0.5 μg) plasmids, respectively, and STE or vehicle was administered 15 h later, and samples were collected 6 h later for and fluorescence quantitative qPCR. The plasmids used in this experiment were purchased from YouBio.

HepG2 2.2.15 cells were seeded at 2.0*10^5^ in Transwell chambers. PMA-induced THP-1 was seeded at 1.2*10^6^ in 24-well plates. After the cells were adhered to the wall, the cells were treated with 1 mg/mL STE, and then co-cultured with the chambers containing HepG2 2.2.15 cells. After 24 h, the cell supernatants were collected, Elisa was used to detect the expression of HBsAg and HBeAg, and THP-1 cells were lysed with Trizol, the transcript level of IFN-β was detected using qPCR (Wu et al., 2024).

### 2.5 Cell viability assay

BMDMs or THP-1 were seeded at a plate density of 1.2*10^6^ cells/mL, and planted in 96-well plates at the corresponding concentration of 0.1 mL per well and left for 12 h. After 12 h, different concentrations of STE (0.25 mg/mL, 0.5 mg/mL, 1 mg/mL, 2 mg/mL, 4 mg/mL, 8 mg/mL, 16 mg/mL, 20 mg/mL) or vehicle were given, and after a further 12 h, 10 μL of CCK-8 was added to all well of the well plates, which were then incubated at 37 °C in an incubator for 50 min. The optical density (OD) values were measured at a wavelength of 450 nm using a Synergy H1 Hybrid Multi-Mode Microplate Reader (BioTek Instruments, Inc.) to assess cell viability (Ren et al., 2022).

### 2.6 Western blotting

The collected whole-cell lysate was heated at 95 °C for 10 min to denature the proteins, followed by brief centrifugation at 12000 rpm for 5 min to remove any insoluble debris. Protein concentration was measured using the BCA Protein Assay Kit (Solarbio,PC0020) according to the manufacturer’s instructions. The samples were loaded onto a 10% SDS-PAGE gel and proteins were separated according to molecular weight at 85 V for 30 min, then adjusted to 140 V and run for an additional 1 h. Proteins were subsequently transferred to PVDF membranes at 450 mA for 3 h using a transfer buffer composed of 25 mM Tris, 192 mM glycine, and 20% methanol. The PVDF membrane was blocked in 5% skim milk powder in TBST (Tris-buffered saline with 0.1% Tween-20) for 1 h at room temperature. The membrane was then washed three times with TBST for 5 min each, followed by incubation with the corresponding primary antibody diluted in 5% skim milk powder in TBST at 4 °C overnight. After incubation, the membrane was washed again three times with TBST for 5 min each, and then incubated with HRP-conjugated secondary antibody (Cell Signaling Technology) diluted 1:5000 in 5% skim milk powder in TBST for 1 h at room temperature. The membrane was washed three times with TBST for 5 min each, and the proteins were visualized using an ECL detection system (Wang et al., 2020).

### 2.7 Quantitative reverse transcription PCR

The RNA Extraction Kit used was the VAMNE Magnetic Universal Total RNA Kit (Vazyme, ROA3301-01), and the Reverse Transcription Kit was the High-Capacity cDNA Reverse Transcription Kit (Applied Biosystems, Catalog Number: 4368813). The reverse transcription was performed according to the manufacturer’s instructions, using a total of 1 μg of RNA for each sample. The RT-qPCR was performed using the SYBR Green qPCR Master Mix (Low ROX) (A304-10, GenStar) on a StepOnePlus Real-Time PCR System (Applied Biosystems). The qPCR cycling conditions were as follows: 95 °C for 10 min, followed by 40 cycles of 60 °C for 30 s and 72 °C for 1 min. The primers used for the target gene were designed to amplify a specific region of the gene, and their sequences were as show in Supplementary Figure S2. Data analysis was performed using the ΔΔCt method, which involves calculating the relative expression levels of the target gene normalized to the reference gene (β-Actin) and relative to a calibrator sample. ence (Yao et al., 2025).

### 2.8 Quantification of cytokines

The levels of IFN-β in cell culture supernatants or mouse serum were determined using the IFN-β ELISA kit (Luex-mifnbv2, InvivoGen). The assay was performed in accordance with the manufacturer’s instructions. Briefly, samples were added to wells of a 96-well plate in triplicate. Following sample addition, the Lucia-Conjugate was introduced to each well. The plate was then incubated at 37 °C for 90 min to allow for the specific binding of IFN-β to the conjugate. After the incubation period, the wells were washed five times with the Washing Buffer provided in the kit to remove any unbound conjugate. Subsequently, the Substrate Solution was added to each well. The luminescence intensity, indicative of the IFN-β concentration, was measured using a luminometer or a multi-mode microplate reader equipped with luminescence detection capabilities. Data were analyzed by comparing the luminescence values of the samples to a standard curve generated using known concentrations of IFN-β. The concentration of IFN-β in the samples was then extrapolated from this standard curve.

The levels of HBsAg and HBeAg were measured following the protocol outlined in the HBeAg ELISA kit instructions, with slight modifications tailored to the detection of HBsAg. Specifically, 5 μL of each test sample, as well as negative and positive controls, were pipetted into the corresponding wells of a microplate. To each well, 50 μL of the enzyme conjugate reagent was added. The plate was then incubated at 37 °C for 30 min to facilitate the binding of the enzyme conjugate to the antigen. After incubation, the wells were washed five times with the washing buffer to remove any unbound enzyme conjugate. Next, 50 μL each of the Chromogen Solution A and Chromogen Solution B were added to the wells. The plate was incubated at 37 °C in the dark for 15 min to allow for color development. Following this, 50 μL of the stop solution was added to each well to terminate the reaction. The absorbance was then measured at a wavelength of 450 nm using a microplate reader to determine the concentration of HBsAg in the samples. The optical density values were used to calculate the antigen levels by comparing them to a standard curve constructed with known concentrations of HBsAg and HBeAg.

The concentration of HBV-DNA was determined using a quantitative PCR-based assay. Initially, 3 μL of nucleic acid release agent was aliquoted into each PCR reaction tube. Subsequently, 3 μL of each test sample, as well as 3 μL of the HBV-negative control, HBV borderline positive control (OT), and HBV quantitative standards (OT) I to IV, were added to their respective PCR tubes. The contents were thoroughly mixed to ensure homogeneity. Following the addition of samples and standards, the samples underwent a lysis procedure to release the nucleic acids. After lysis, 32 μL of PCR mix was added to each tube. The tubes were then centrifuged to ensure all components were at the bottom of the tubes, and were subsequently loaded onto the PCR machine for amplification. The presence and quantity of HBV-DNA were determined by analyzing the fluorescent signal generated during the PCR process using a real-time PCR instrument. The data were analyzed by comparing the cycle threshold (Ct) values of the samples to a standard curve constructed with known concentrations of HBV-DNA.

### 2.9 Flow cytometry

The ratios of immune cells and interferon in mouse spleen tissues after STE and ETV interventions were examined using flow cytometry. After rinsing the collected spleen tissues with PBS, the tissues were dissociated in RPMI-1640 medium containing 2% FBS (Fetal Bovine Serum). The cell suspension was then filtered through a 200 μm nylon mesh to obtain a single-cell suspension. Subsequently, Zombie NIR™ Fixable Viability Kit 423105, 1:5000, BioLegend) was added to the cell suspension and centrifuged to lyse and remove erythrocytes. The remaining cells were washed with PBS. The cells were fixed with 4% Paraformaldehyde (G1101-500ML, Servicebio) for 10 min at room temperature. After fixation, the cells were washed with PBS and stained with the appropriate antibody for 50 min at 4 °C in the dark. Following incubation, the cells were washed with PBS to remove unbound antibody. Finally, all samples were analyzed by flow cytometry, and the data were processed and analyzed using FlowJo 10.8.1 software (FlowJo LLC).

### 2.10 Statistical analysis

Statistical analysis was performed by GraphPad 8.0, and all data were showed as Mean ± SD, when p < 0.05, statistical differences were considered to exist. Statistical analyses were performed on multiple samples using one-way ANOVA with Dunnett’s *post hoc* test.

## 3 Results

### 3.1 STE promotes cGAS-STING pathway activation in BMDMs and THP-1

**FIGURE 1:**
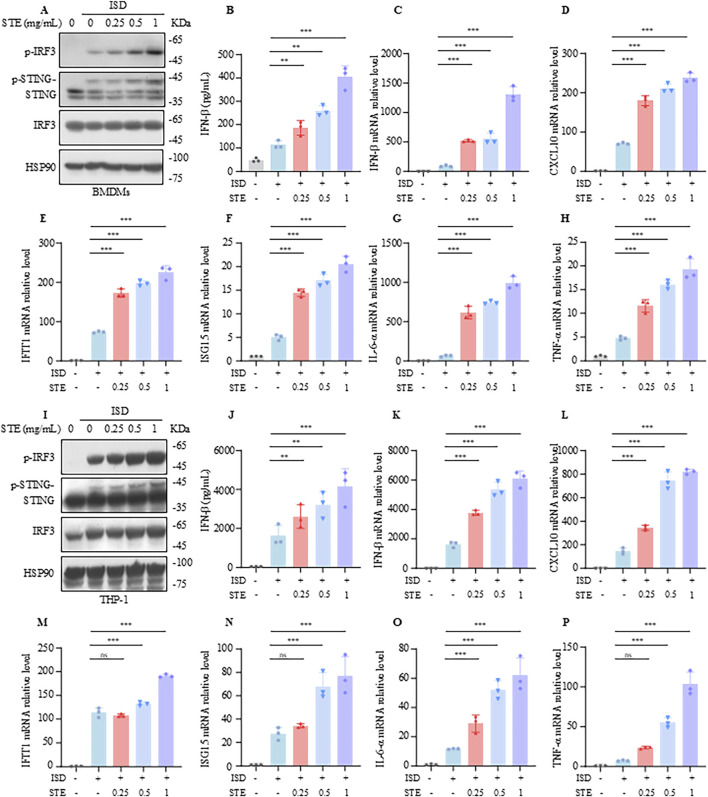
STE promotes cGAS-STING pathway activation in BMDMs and THP-1. **(A)** BMDMs were first treated with STE or vehicle for 1 h, followed by transfection with ISD for 2.5 h. Cell lysates were collected and assayed for the expression levels of the proteins shown therein. **(B)** BMDMs were first treated with STE or vehicle for 1 h and then transfected with ISD for 8 h, in which IFN-β levels were detected. **(C–H)** BMDMs were first treated with STE or vehicle for 1 h, then transfected with ISD for 4 h. Cells were lysed with Trizol and IFN-β, CXCL10, IFIT1, ISG15, IL-6 and TNF-α levels were detected in them. **(I)** PMA-primed THP-1 were first treated with STE or vehicle for 1 h, followed by transfection with ISD for 2.5 h. Cell lysates were collected and assayed for the expression levels of the proteins shown therein. **(J) **PMA-primed THP-1 were first treated with STE or vehicle for 1 h and then transfected with ISD for 8 h, in which IFN-β levels were detected. **(K–P)** PMA-primed THP-1 were first treated with STE or vehicle for 1 h, then transfected with ISD for 4 h. Cells were lysed with Trizol and IFN-β, CXCL10, IFIT1, ISG15, IL-6 and TNF-α levels were detected in them. Data in **(B-H)**, **(J-P)** are expressed as mean ± SD from three biological replicates. Statistical analyses were performed on multiple samples using one-way ANOVA with Dunnett’s *post hoc* test. *p < 0.05, **p < 0.01 and ***p < 0.001 vs. the control, ns, not significant.

In this study, we explored the impact of STE on the activation of the cGAS-STING pathway. We initially assessed the effect of STE at varying concentrations (0–20 mg/mL) on the viability of BMDMs and THP-1 cells to establish a safe concentration range. Our findings indicated that STE concentrations up to 2 mg/mL did not affect cell viability, whereas concentrations of 4 mg/mL or higher began to exhibit cytotoxic effects (Supplementary Figure S2A,B). Subsequently, BMDMs were pretreated with STE at concentrations of 0.25, 0.5, and 1 mg/mL for 1 hour, followed by transfection with ISD to activate the cGAS-STING pathway. We then measured the expression levels of p-IRF3, p-STING, STING, and IRF3 using immunoblotting. The results demonstrated that STE promoted the phosphorylation of IRF3 and STING in a concentration-dependent manner (Figure 1A) and enhanced the production of IFN-β induced by ISD (Figure 1B). qPCR analysis further revealed that STE significantly upregulated the expression of IFN-β, CXCL10, IFIT1, ISG15, IL-6, and TNF-α (Figures 1C–H), indicating an enhancement of cGAS-STING activation in BMDMs. Similarly, the activating effect of STE on the cGAS-STING pathway in THP-1 cells was investigated. The results showed that STE promoted the phosphorylation of IRF3 and STING, increased IFN-β production, and elevated the transcription levels of IFN-β, CXCL10, IFIT1, ISG15, IL-6, and TNF-α in THP-1 cells stimulated with ISD (Figures 1I–P). Collectively, our data indicate that STE significantly enhances cGAS-STING activation and the induction of interferon-stimulated genes and inflammatory cytokines in both BMDMs and THP-1 cells.

### 3.2 STE does not affect cytoplasmic RNA-Induced increases in interferon levels

**FIGURE 2:**
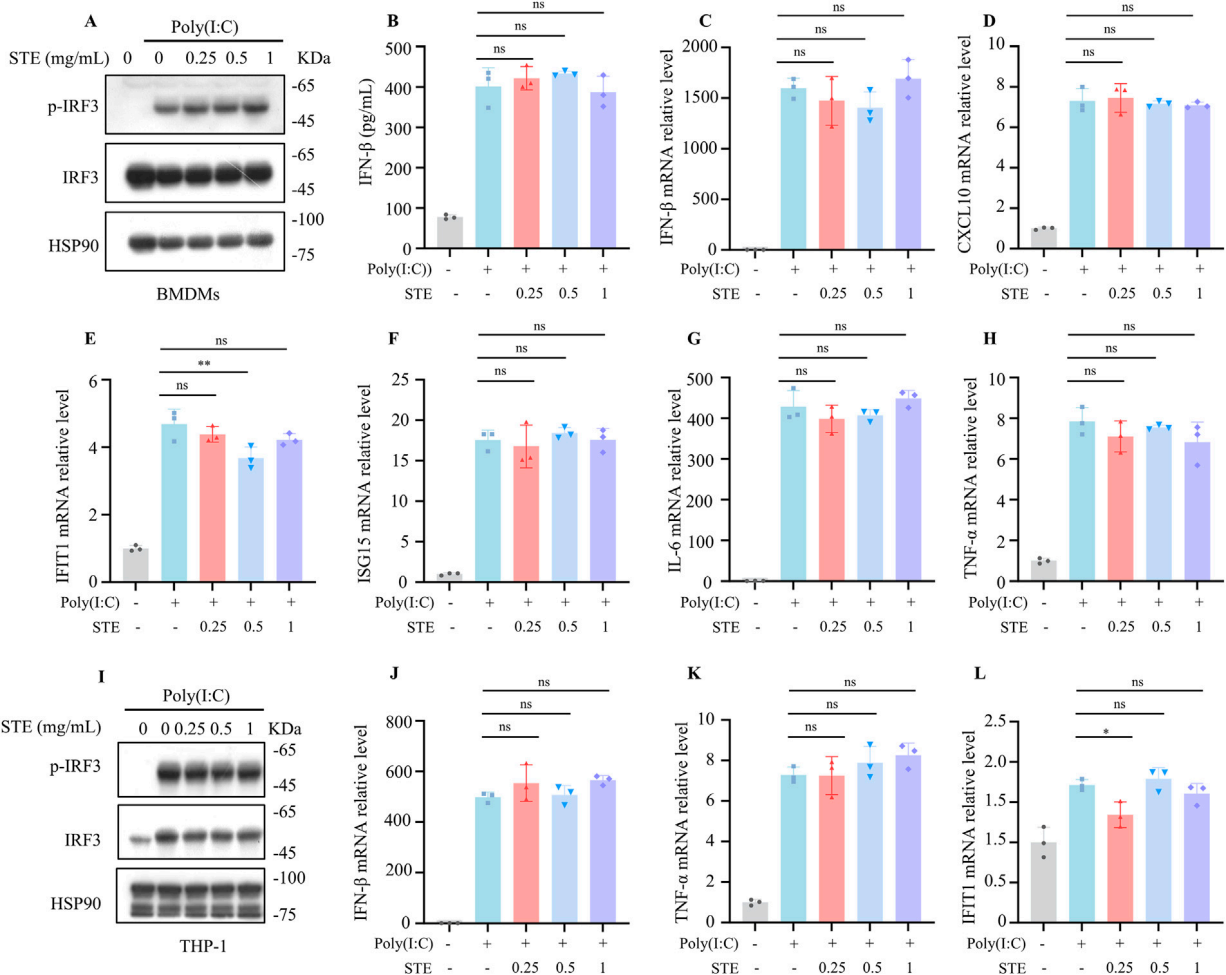
STE does not affect cytoplasmic RNA-induced increases in interferon levels. **(A)** BMDMs were first treated with STE or vehicle for 1 h, followed by transfection with Poly(I:C) for 2.5 h. Cell lysates were collected and assayed for the expression levels of the proteins shown therein. **(B)** BMDMs were first treated with STE or vehicle for 1 h and then transfected with Poly(I:C) for 8 h, in which IFN-β levels were detected. **(C–H)** BMDMs were first treated with STE or vehicle for 1 h, then transfected with Poly(I:C) for 4 h. Cells were lysed with Trizol and IFN-β, CXCL10, IFIT1, ISG15, IL-6 and TNF-α levels were detected in them. **(I)** PMA-primed THP-1 were first treated with STE or vehicle for 1 h, followed by transfection with Poly(I:C) for 2.5 h. Cell lysates were collected and assayed for the expression levels of the proteins shown therein. **(J–L)** PMA-primed THP-1 were first treated with STE or vehicle for 1 h, then transfected with Poly(I:C) for 4 h. Cells were lysed with Trizol and IFN-β, TNF-α and IFIT1 and levels were detected in them. Data in **(B-H,J-L)** are expressed as mean ± SD from three biological replicates. Statistical analyses were performed on multiple samples using one-way ANOVA with Dunnett’s *post hoc* test. *p < 0.05, **p < 0.01 and ***p < 0.001 vs. the control, ns, not significant.

We next sought to determine whether STE influences the RIG-I pathway, another crucial antiviral signaling mechanism that detects intracellular pathogenic RNA. To investigate the potential regulatory effects of STE on the RIG-I pathway, we established an activation model using Poly(I:C) transfection. BMDMs were treated with STE for 1.5 h, followed by transfection with Poly(I:C) for an additional 2.5 h. Our findings indicated that STE did not alter the levels of p-IRF3, the amount of IFN-β in the supernatant, or the expression of downstream interferon-stimulated genes (Figures 2A–H). Similarly, in THP-1 cells, STE had no impact on Poly(I:C)-induced pathway activation or the upregulation of gene expression (Figures 2I–L). These results suggest that STE does not influence the activation of the RIG-I pathway.

### 3.3 STE enhances cGAS-STING pathway activation via promoting 2′3′-cGAMP synthesis

**FIGURE 3:**
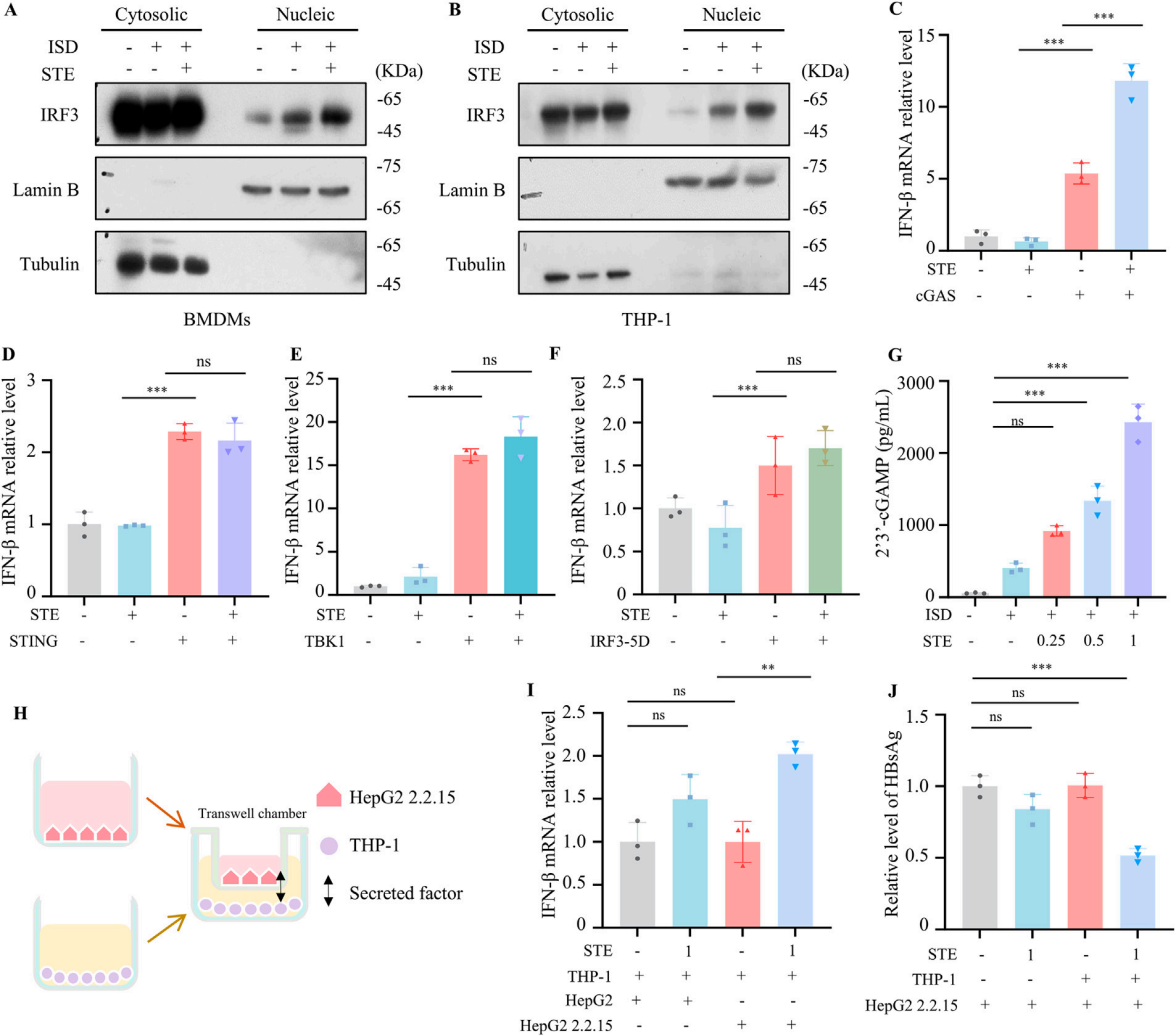
STE promotes cGAS-STING pathway activation via facilliating 2′3′-cGAMP synthesis **(A,B)** BMDMs or THP-1 were first treated with STE for 1 h and then with ISD for 2.5 h. Subsequently, cell membranes and nuclei were isolated and the amount of IRF3 protein in them was detected. **(C–F)** The cGAS, STING, TBK1 or IRF3-5D plasmids were transfected into HEK-293 cells for 18 h, then STE (1 mg/mL) was administered, and the expression level of IFN-β in them was detected by qPCR after 6 h (n = 3). **(G)** BMDMs were treated with STE for 1.5 h followed by ISD for 1.5 h and 2′3′-cGAMP levels in the samples were detected using ELISA kits (n = 3). **(H–J)** THP-1 cells inoculated in 24-well plates were first treated with STE. Transwell chambers inoculated with HepG2.2.2.15 cells were placed in well plates containing THP-1 cells, and after 24 to detect the HBsAg and IFN-β levels (n = 3). Data are expressed as mean ± SD from three biological replicates. Statistical analyses were performed on multiple samples using one-way ANOVA with Dunnett’s *post hoc* test. *p < 0.05, **p < 0.01 and ***p < 0.001 vs. the control, ns, not significant.

We further explored the mechanism by which STE regulates the cGAS-STING pathway. Our data demonstrated that STE could promote the translocation of IRF3 to the nucleus, confirming its effect on cGAS-STING activation (Figures 3A,B). To investigate the mechanism of STE in promoting cGAS-STING pathway activation, HEK293 cells were transfected with plasmids expressing cGAS, STING, TBK1, or IRF3-5D, which is a constitutively active mutant of IRF3. After administration of STE, the expression of IFN-β was detected by qPCR. The results indicated that STE promoted IFN-β expression induced by cGAS overexpression but had no significant effect on STING, IRF3-5D, and TBK1-induced increases in IFN-β expression (Figures 3C–F). This suggests that STE may act upstream of STING to promote cGAS-STING activation. We next examined the effect of STE on the production of 2′3′-cGAMP, which is synthesized by cGAS upon DNA stimulation. The results showed that 2′3′-cGAMP synthesis could be dose-dependently enhanced by STE in both BMDMs and THP-1 cells (Figure 3G; Supplementary Figure S3A). These findings indicate that STE promotes cGAS-STING pathway activation by facilitating the synthesis of 2′3′-cGAMP mediated by cGAS.

### 3.4 STE shows anti-HBV effect *in vitro*

Given the crucial role of the cGAS-STING pathway in controlling infections of multiple viruses, including HBV, we investigated the inhibitory effect of STE on HBV *in vitro*. We established a co-culture system to verify the anti-HBV activity of STE. Transwell chambers seeded with HepG2 2.2.15 cells were placed on top of 24-well dishes containing THP-1 cells treated with STE or vehicle. After 24 h, the levels of HBeAg, HBsAg, and IFN-β were detected. The results demonstrated that STE significantly increased the expression of IFN-β when added to the THP-1 and HepG2.2.15 co-culture system (Figure 3H). Additionally, STE effectively reduced the levels of HBeAg and HBsAg (Figures 3I,J, S3B). Specifically, the reduction in HBsAg levels was 50%, respectively, compared to the control group. However, STE had no significant effect on HBsAg levels when directly applied to HepG2.2.15 cells, suggesting that STE enhances cGAS-STING activation and IFN production in THP-1 cells, thereby indirectly suppressing HBsAg and HBsAg level in HepG2.2.15 cells.

### 3.5 STE suppresses HBV replication and promotes cGAS-STING pathway activation in HBV model mice

**FIGURE 4:**
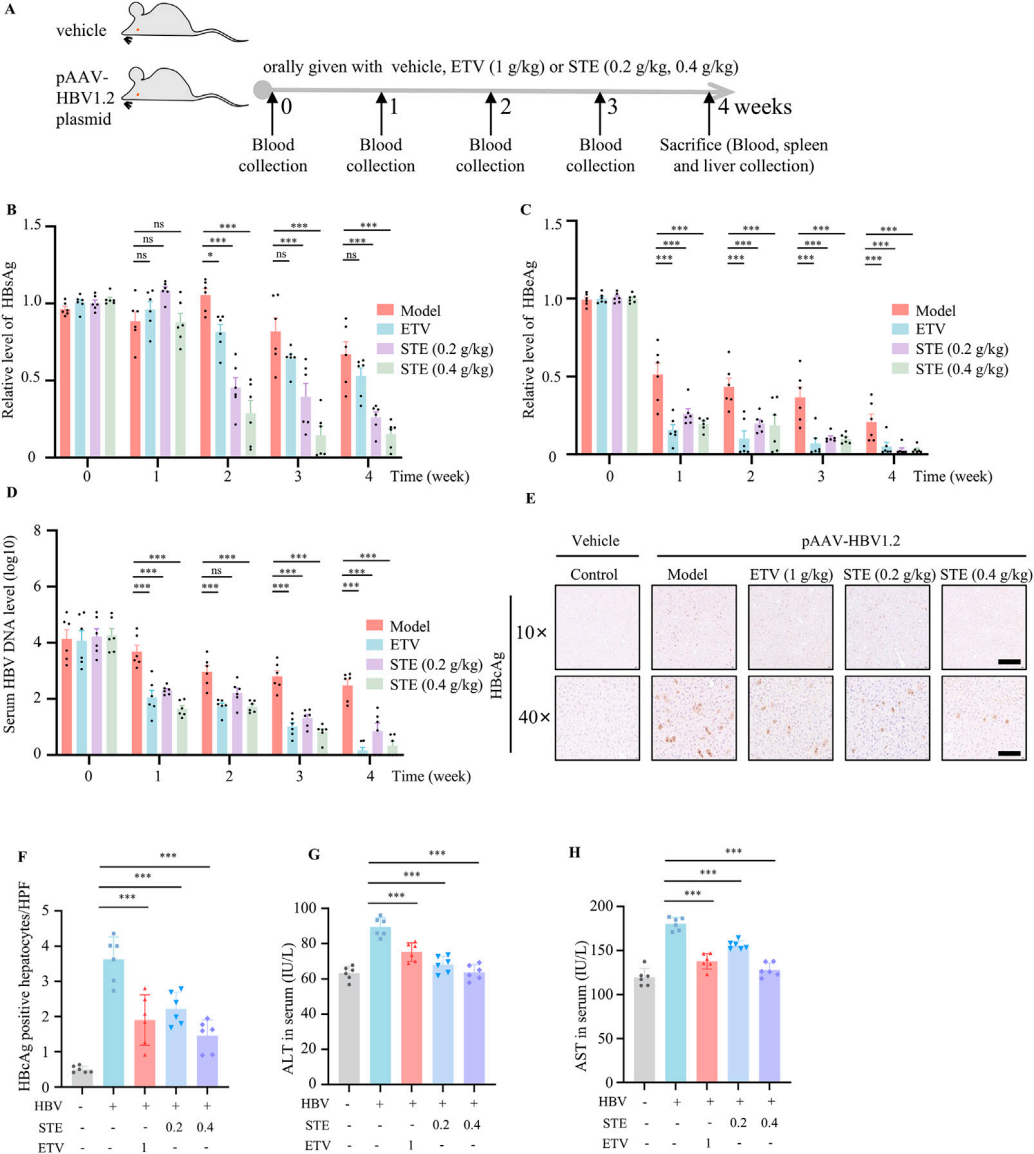
STE restrains HBV replication and promotes cGAS-STING pathway activation. **(A–D)** The HBV model was constructed by injecting 20 μg of pAAV/HBV1.2 plasmid into mice, and STE or ETV was given daily, while mice in the blank group were given saline **(A)**. The serum levels of HBsAg **(B)**, HBeAg **(C)** and HBV DNA **(D)** in mice were detected weekly. **(E,F)** Immunohistochemical method to detect the expression level of HBcAg in fractional liver tissues, scale bars: 250 μm (top row); 50 μm (bottom row) (n = 6). **(G,H)** Serum levels of ALT and AST were measured by ELISA kits and physiological and biochemical kits (n = 6). Data are presented as mean ± SD (n = 6 per group) one-way ANOVA and Dunnett’s *post hoc* test were used to assess the differences of multiple groups, ***p < 0.001 vs. the model group, ns, not significant.

Next we tested if SET inhibited HBV replication *in vivo*. The HBV mice model was constructed by hydrodynamic injection of pAAV-HBV1.2 plasmid, the mice were divided into 4 groups: normal saline group, STE (0.2 g/kg) group, STE (0.4 g/kg) group, or ETV (1 g/kg) (Figure 4A). The results found that HBsAg levels in serum of mice decreased after administration of STE, the effect was even better than ETV (Figure 4B). While serum HBeAg and HBV DNA level showed downregulation after administration of STE or ETV, the therapeutic effect of high dose of STE was similar to that of ETV group (Figures 4C,D). Meanwhile, we performed immunohistochemical staining of the collected liver tissues to detect the number of HBcAg-positive hepatocytes, there were fewer positive cells in mice of STE or ETV group than the model group (Figures 4E,F). These results showed that STE can significantly suppress HBV replication in mice. Next, we examined the expression levels of ALT and AST in the serum of mice (Figures 4G,H). The data found that after the administration of STE, the levels of ALT and AST appeared to be reduced compared to the model group, which indicated that hepatic STE could improve the liver histopathology in mice.

### 3.6 STE increases the level of IFN-γ in spleen tissue of HBV mice

**FIGURE 5:**
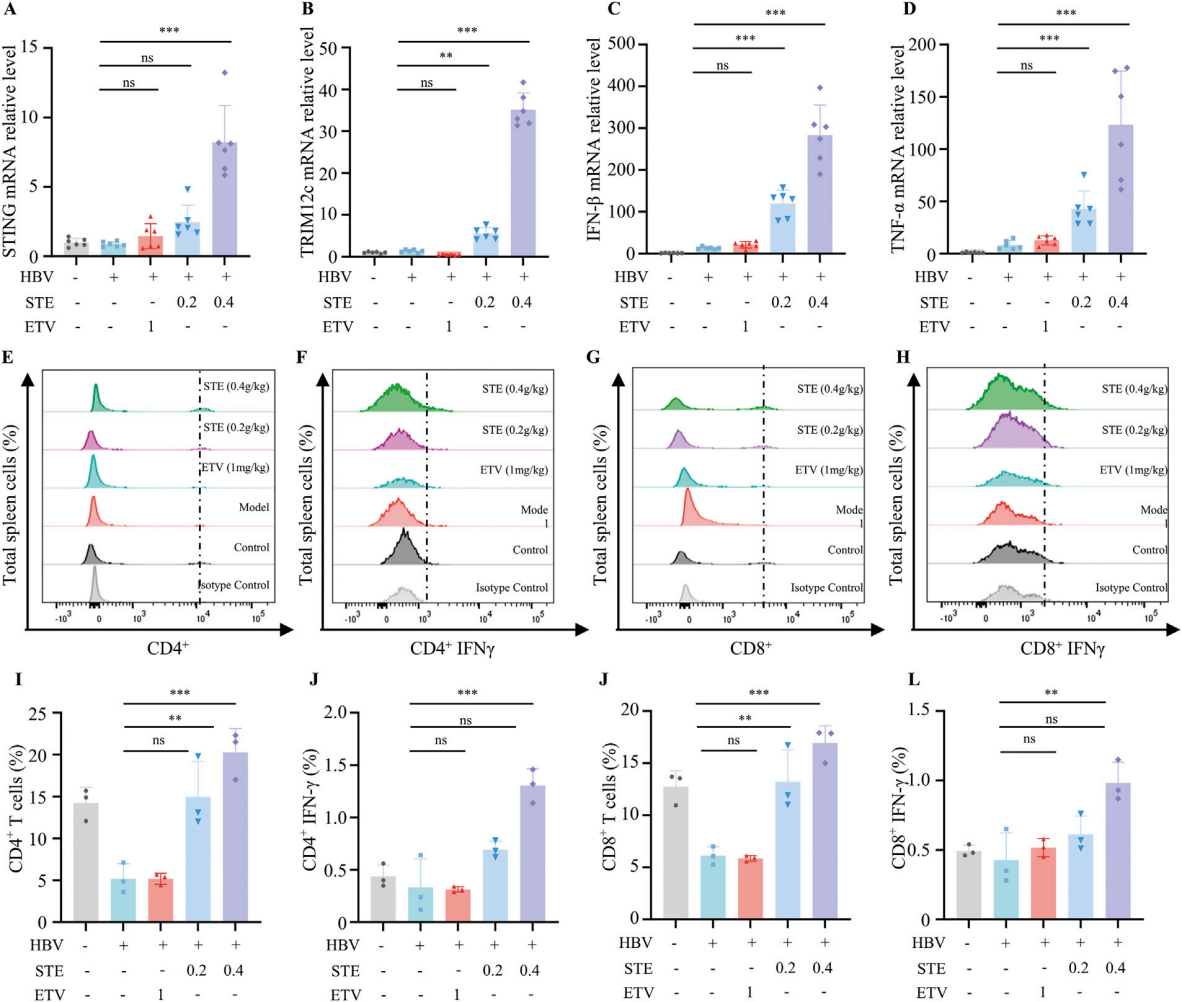
STE increases IFN-γ levels in spleen tissue of HBV mice. **(A–D)** The collected mouse liver tissues were homogenized, and the total RNA was lysed and extracted by Trizol. qPCR was used to detect the levels of STING **(I)**, TRIM12c **(J)**, IFN-β **(K)**, and TNF-α **(L)** in the liver tissues (n = 6). **(E–H)** Flow cytometry was performed to analyze the levels of CD4^+^, CD8^+^ T cells as well as IFN-γ^+^CD4, IFN-γ^+^CD8^+^ cells in spleen tissues (n = 3). **(I–L)** Statistical analysis of the frequency of CD4^+^, CD8^+^ T cells as well as IFN-γ^+^CD4, IFN-γ^+^CD8^+^ (n = 3). Data are presented as mean ± SD, Statistical analyses were performed on multiple samples using one-way ANOVA with Dunnett’s *post hoc* test. *P < 0.05, **P < 0.01, ***P < 0.001, ns: not significant.

We next assessed whether STE promotes the expression of key genes involved in cGAS-STING pathway activation. Gene expression of STING, IFN-β, TNF-α and TRIM12C in liver tissue was significantly induced in mice treated with Ste (Figures 5A–D) Specifically, the expression of STING, IFN-β, TNF-α, and TRIM12C genes was significantly increased in Ste liver tissue in the high-dose group compared with the control group, indicating that Ste enhanced the activation of cGAS-STING signaling *in vivo*. We also examined the frequency of CD4^+^ and CD8^+^ T cells in the spleen of HBV mice. In our study, we observed a significant increase in the frequency of CD4^+^ T and CD8^+^ T cells in the STE groups compared with the control group (Figures 5E,G). The percentage increase was 35% for CD4^+^ T cells and 40% for CD8^+^ T cells. Additionally, we evaluated the level of IFN-γ in CD4^+^ and CD8^+^ T cells, and the frequency of IFN-γ^+^ CD4^+^ and IFN-γ^+^ CD8^+^ T cells. The frequency of IFN-γ^+^ CD4^+^ and IFN-γ^+^ CD8^+^ T cells in the STE group was increased by 3-fold and 2-fold, respectively, compared to the model group (Figures 5F,H,I). These results suggest that STE enhances the activation of both CD4^+^ and CD8^+^ T cells in HBV mice.

Taken together, these results suggest that STE inhibits HBV replication, improves liver histopathology, and promotes cGAS-STING pathway activation *in vivo*. These results suggest that the inhibition of HBV by STE may be associated with the promotion of cGAS-STING signaling activation.

## 4 Discussion

Our study shows that STE specifically promotes cGAS-STING activation and the expression of interferon, the mechanism may be related with the facilitation of 2′3′-cGAMP synthesis. In addition, STE inhibits HBV replication, attenuates pathological liver injury, enhances the antiviral immunity in HBV mice, suggesting that STE has a good antiviral potential.

In our study, we established an *in vitro* model of cGAS-STING pathway activation in BMDMs and THP-1 cells to investigate the effect of STE (Wang Y. et al., 2024). The results suggested that STE could dose-dependently promote the phosphorylation of STING and IRF3 proteins in a safe concentration range, the expression of IFN-β in supernatants and the production of IFN-β, CXCL10, IFIT1, ISG15, IL-6 and TNF-α in cells. It was confirmed that the activation of RIG-I pathway caused by aberrant RNA in cells can also have to the release of type I interferon and pro-inflammatory cytokines (Zheng et al., 2022), and in order to demonstrate whether STE specifically inhibits the cGAS-STING, we evaluated the effect of STE in the presence of Poly(I:C). The results showed that STE had no effect on the RIG-I pathway, thus STE specifically promoted the activation of the cGAS-STING.

We transfected plasmids encoding proteins involved in cGAS-STING pathway into HEK293 cells, and found that STE only promoted the elevation of the IFN-β in HEK293 cells transfected with cGAS, but had no effect on that in HEK293 cells overexpressing other proteins, demonstrating STE may affect cGAS function. Once recognizing DNA, cGAS becomes activated and catalyzes ATP and GTP into 2′-3′ cGAMP (Mu et al., 2025), our result also showed STE could dose-dependently enhance the amount of 2′-3′ cGAMP in cells stimulated with ISD. Our data demonstrated that STE may affect cGAS function and facilitate 2′-3′ cGAMP by cGAS. However, if STE enhances the binding of DNA to cGAS or regulates its enzymatic activity awaits further study.

cGAS-STING pathway activation has been reported to restrain HBV replication, though the expression of STING is relatively low in hepatocytes, STING agonists could effectively induce STING activation in macrophages, leading to production of IFN and other cytokines, which in turn effectively suppresses HBV replication in hepatocytes (Lau et al., 2020). In our study, we established a co-culture system, THP-1 cells were co-cultured with HepG2.2.15 cells, STE effectively induced IFN-βexpression and inhibited HBV replication, however STE alone did not affect HBV replication in HepG2.2.15 cells. The data suggested that STE indirectly suppressed HBV in hepatocytes via promoting cGAS-STING pathway activation and IFN-βproduction in macrophages.

We also established an HBV model in mice, the results showed that STE reduced the levels of HBeAg, HBcAg, HBsAg, and HBV DNA, and improved liver pathology in mice. It is noteworthy that clinical studies have shown that the effect of ETV in lowering HBsAg is not prominent, whereas in our *in vivo* experiments as well as *in vitro* co-culture experiments, STE was able to lower the HBsAg level, which is an advantage of STE, and the therapeutic efficacy of high-dose STE was the same as that of ETV in reducing the levels of HBeAg, HBcAg, and HBV DNA. Previous literature has found that Schisandra chinensis can also inhibit HBV replication by promoting activation of the cGAS-STING pathway (Zhao J. et al., 2023), which is consistent with the findings of this study. This suggests that activating cGAS-STING pathway may be an effective modality for anti-HBV and holds great promise for the future treatment of chronic hepatitis B. Although STE-boosted cGAS-STING signalling potently curtailed HBV replication in our model, sustained hyper-activation of this axis could generate high systemic levels of IFN-β and IL-18, raising concerns about cytokinaemia-related adverse events. Moreover, because chronic hepatitis B is fundamentally an immune-mediated disorder, excessive intra-hepatic STING activity might amplify necro-inflammatory injury, triggering ALT flares or accelerating fibrosis (Ito et al., 2019; Dickson, 2016). Our present data revealed no elevation of transaminases after 4 weeks of treatment; however, prolonged high-dose exposure or combination with other immunotherapeutic regimens could tilt the balance toward hepatocyte damage, which is a concern for future research.

Notably, STE can inhibit HBV replication by modulating the natural immune pathway rather than directly inhibiting HBV, which is a new mechanism by which STE inhibits HBV. Unlike previous ethnopharmacological studies that focused primarily on the hepatoprotective or anti-fibrotic effects of STE, the present work integrates traditional usage with mechanistic dissection of innate immunity, thereby extending the scientific basis of this medicinal plant from empirical experience to a defined molecular target—the cGAS–STING axis. This alignment underscores the central aim of ethnopharmacology: bridging traditional remedies with modern therapeutic pathways and providing a mechanistic rationale for the continued clinical development of STE as an adjunct immunomodulator in chronic hepatitis B (Liu et al., 2022). However, this study did not use STING knockout mice to validate the mechanism of action of STE against HBV.

In conclusion, in this work, we found that STE could enhance cGAS-STING activation by facillating 2′-3′ cGAMP production, it shows obviously inhibitory effect on HBV replication, STE may be a promising potential drug for the therapy of chronic hepatitis B and also other viral infectious diseases.

## Data Availability

The datasets presented in this study can be found in online repositories. The names of the repository/repositories and accession number(s) can be found in the article/Supplementary Material.

## Abbreviations

STE, Sophorae tonkinensis Radix et Rhizoma extract; ETV, entecavir; HBV, hepatitis B virus; HBsAg, hepatits B surface antigen; HBeAg, hepatitis Be antigen; HBcAg, hepatitis B core antigen; cGAS, cyclic GMP-AMP synthase; STING, stimulator of interferon genes; cGAMP, cyclic-GMP-AMP; IRF3, interferon regulatory factor 3; BMDMs, bone marrow derived macrophage; THP-1, human Acute Monocytic Leukemia Cells; ISD, interferon stimulatory DNA; Poly(I:C), polyinosinic-polycytidylic acid; IFN β, interferon β; TNF α, tumour necrosis factor-alpha; IL-6, interleukin 6; CXCL10, C-X-C motif chemokine ligand 10; IFIT1, interferon-induced protein with tetratricopeptide repeats 1; ISGs, interferon-stimulated genes; TRIM12c, tripartite motif 12c; PMA, 12-O-tetradecanoylphorbol-13-acetate.
