# Supplementary figures and images for: Sophora tonkinensis enhances activation of cGAS-STING pathway and restrains HBV replication
Source: Front Pharmacol. 2025 Oct 13;16:1630460. doi: 10.3389/fphar.2025.1630460 (PMC12554753; doi:10.3389/fphar.2025.1630460)

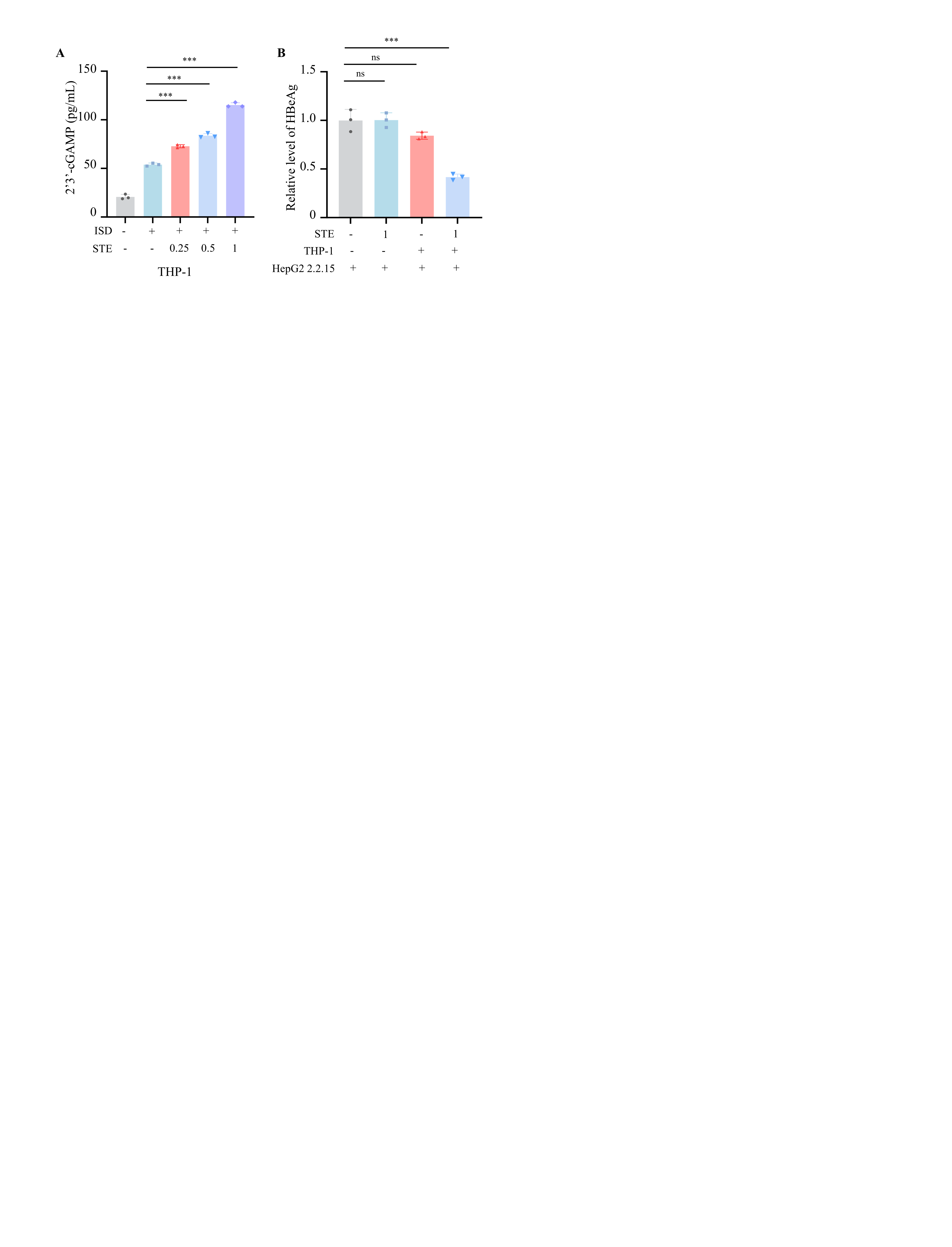

Supplement: Supplementary file 1 [file Image3.tif]

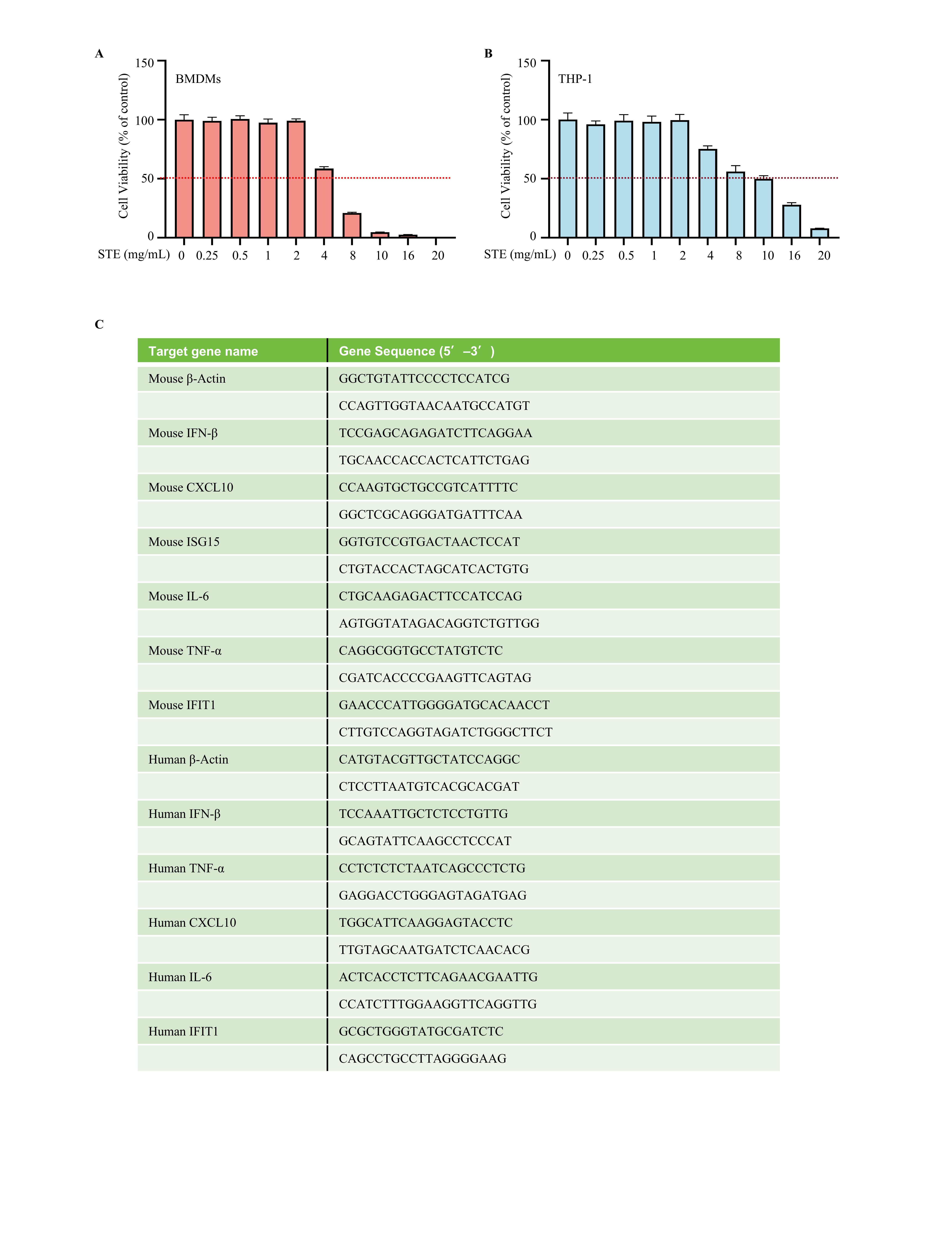

Supplement: Supplementary file 2 [file Image2.tif]

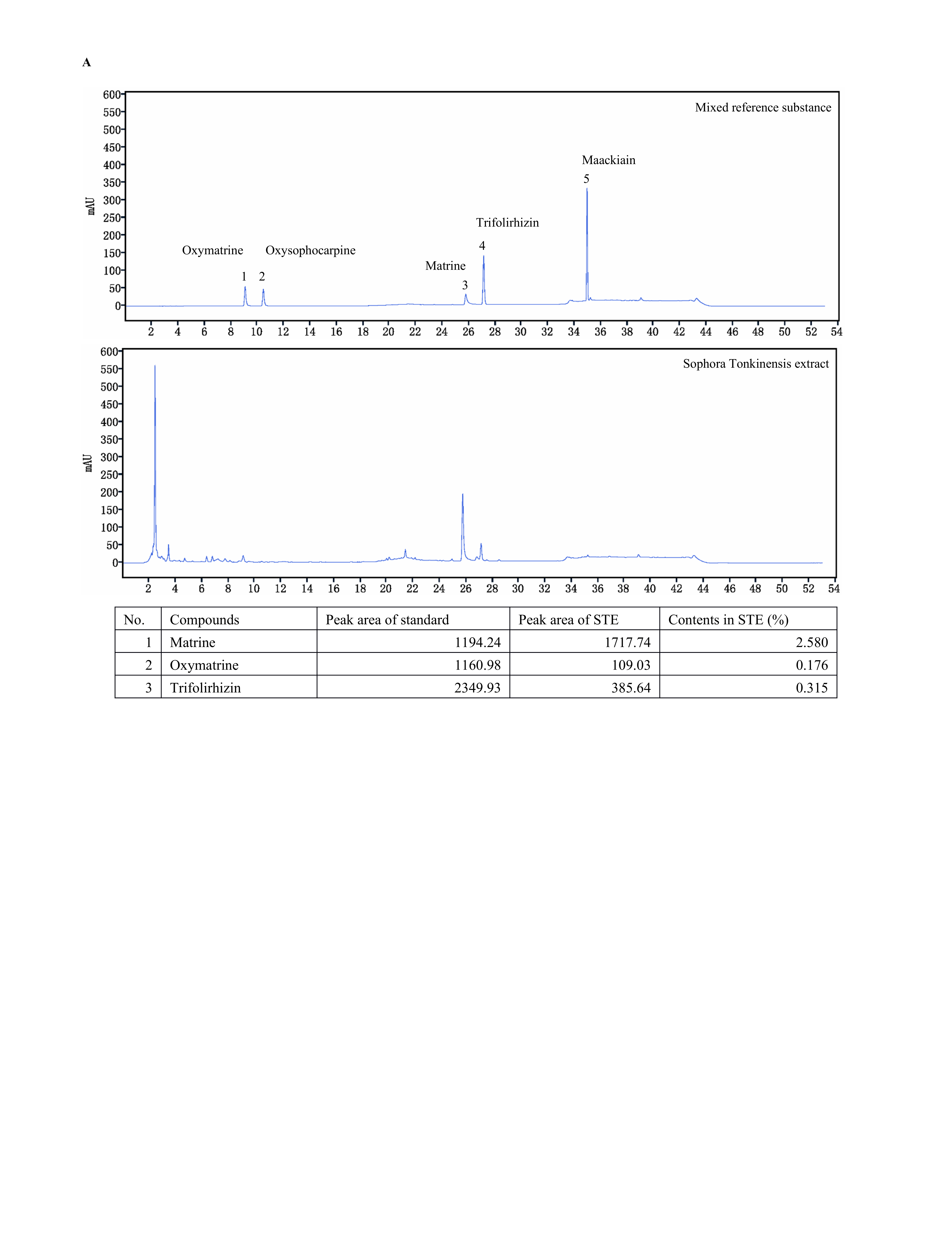

Supplement: Supplementary file 3 [file Image1.tif]
